# A cluster randomized controlled trial on the effects and costs of advance care planning in elderly care: study protocol

**DOI:** 10.1186/s12877-015-0087-z

**Published:** 2015-07-22

**Authors:** Ida J. Korfage, Judith A.C. Rietjens, Anouk Overbeek, Lea J. Jabbarian, Pascalle Billekens, Bernard J. Hammes, Ellen Hansen-van der Meer, Suzanne Polinder, Johan Severijnen, Siebe J. Swart, Frederika E. Witkamp, Agnes van der Heide

**Affiliations:** Department of Public Health, Erasmus Medical Center, Rotterdam, The Netherlands; Laurens, Rotterdam, The Netherlands; Gundersen Lutheran Medical Foundation, La Crosse, WI USA

**Keywords:** Advance care planning, Advance directives, Respecting choices, Elderly care, Cost-effectiveness, Quality of life, Patient activation

## Abstract

**Background:**

Currently, health care and medical decision-making at the end of life for older people are often insufficiently patient-centred. In this trial we study the effects of Advance Care Planning (ACP), a formalised process of timely communication about care preferences at the end of life, for frail older people.

**Methods/Design:**

We will conduct a cluster randomised controlled trial among older people residing in care homes or receiving home care in the Netherlands. The intervention group will receive the ACP program Respecting Choices® in addition to usual care. The control group will receive usual care only. Participants in both groups will fill out questionnaires at baseline and after 12 months. We hypothesize that ACP will lead to better patient activation in medical decision making and quality of life, while reducing the number of medical interventions and thus health care costs. Multivariate analysis will be used to compare differences between the intervention group and the control group at baseline and to compare differences in changes after 12 months following the inclusion.

**Discussion:**

Our study can contribute to more understanding of the effects of ACP on patient activation and quality of life in frail older people. Further, we will gain insight in the costs and cost-effectiveness of ACP. This study will facilitate ACP policy for older people in the Netherlands.

**Trial registration:**

Nederlands Trial Register: NTR4454.

## Background

The number of Europeans over 65 years of age will double in the next 50 years. To enable countries to successfully manage the dynamics and health care costs of their ageing populations, the WHO has proposed an “active ageing approach”, based on the United Nations Principles of independence, participation, dignity, care and self-fulfillment [1]. It targets government decision-makers at all levels, the nongovernmental sector and the private sector, all of whom are responsible for the formulation of policies and programs on ageing. It focuses on the activation of older people and is aimed at timely recognition and consideration of people’s health care preferences and needs to enable strategic planning and decision-making by older people themselves. Communication about people’s needs and preferences is typically postponed until acute events necessitate short-term medical decision-making. Sharp et al. showed that the majority of frail, older people would like to get the opportunity to timely discuss end-of life care. However, most of them do not have this opportunity [2].

Advance Care Planning (ACP) is a formalised process of communication between patients, their relatives and professional caregivers about patients’ health preferences, goals and choices [3]. Its central aim is to activate people to participate in decision-making about their health care and to raise awareness of the need to anticipate future deterioration of health. Patients are encouraged to appoint a surrogate decision-maker, and to document their wishes about their preferred care in an advance directive. In this way, written advance directives extend the autonomy of patients to a phase when they are incompetent [4].

Several studies have assessed the outcomes of ACP programs. In der Schmitten et al. studied the effects of implementing a regional ACP program in German nursing homes and concluded that the implementation led in many cases to the completion of advance directives with potential relevance to medical decision-making [5]. Some reviews showed that ACP is associated with better patient outcomes. In a recent systematic review, Brinkman-Stoppelenburg et al. showed that some ACP programs are associated with a reduction in futile measures and unnecessary hospitalizations [6]. The programs contributed to better communication between patients and health care professionals and higher quality of life of both patients and their relatives. Furthermore, extensive ACP interventions seemed to increase compliance with patient wishes and satisfaction with care more than just the completion of advance directives. According to the review of Brinkman-Stoppelenburg et al. extended programs such as e.g. the Respecting Choices program are the most promising ACP programs [6]. In another systematic review, Houben et al. showed that ACP programs facilitated completion of advance directives and end-of-life discussions between patients and health care professionals. The programs improved concordance between preferred and delivered care and potentially improve other outcomes as well, such as quality of communication [7].

Overall, we can conclude that ACP programs have beneficial effects on several patient outcomes. However, evidence on the effects of ACP programs in care homes and in community dwelling older people is scarce [6, 7]. Furthermore, few studies concerned a randomized controlled trial, although this is the most preferred method of studying effects of interventions in health care. Finally, most of the studies were performed in the US. Previous research showed that end-of-life decision-making varies largely between countries [8]. It is unknown to what extent results from the US can be generalized to European countries like the Netherlands, given the differences in health care systems.

Also, research on the costs of ACP is limited, although the cost-effectiveness of health care interventions at the end of life is an important issue due to the fact that end-of-life care is often expensive. While health care costs in an average life year have been found to amount to 1100 € per person, costs per last year of life were 13,5 times higher and approximated 14,900 € per person according to Polder et al. [9]. Molloy et al. assessed the costs of an ACP program in a randomized controlled trial in Ontario, Canada. Intervention nursing homes reported fewer hospitalizations per resident, a lower hospital length of stay ensuing reduced hospital care costs and lower total health care costs than control nursing homes [10]. We are not aware of any formal cost-effectiveness studies on ACP programs in care homes or nursing homes in Europe.

We will perform the first cluster randomized controlled trial on the effects of ACP in frail, older people in the Netherlands. The main objective of this project is to assess the effects, the costs and the cost-effectiveness of ACP. Participants in the intervention group will be offered the Respecting Choices program, one of the most promising ACP programs (see below for further information about the program) [6, 11]. We focus on older people living in care homes or living at home and receiving home care. We hypothesize that ACP will lead to better patient activation in medical decision making and quality of life in the intervention group compared to the control group, while reducing futile interventions and thus health care costs.

## Methods/Design

### Study design

We will conduct a cluster Randomized Controlled Trial (RCT) among older people living in care homes or at home receiving home care, following the CONSORT guidelines. The clusters will consist of neighbourhoods. In the Netherlands, standardized household incomes have been calculated per neighbourhood. We will order neighbourhoods according to these standardized incomes to control for differences in income per study arm, since income is related to health [12]. Neighbourhoods will be randomized per set of two neighbourhoods with comparable household incomes to either the control or intervention condition.

### Study population

The study population consists of older people living at home and receiving home care or residing in one of 16 participating care homes of Laurens, a care organisation in Rotterdam, the Netherlands. Residents of care homes generally suffer from one or more chronic conditions and 32 % are totally dependent on others for daily care needs [13]. Their average life expectancy is 3,7 years, and their health condition often involves important health care decisions in a relatively short time frame.

### Inclusion criteria

In order to be eligible to participate in this study, an older person must meet all of the following criteria:

1. ≥75 years of age;
2. Mentally competent, as measured by judgement of caregiver and a Mini–Mental State Examination (MMSE score > 16 [14];
3. Fluent in Dutch;
4. Being frail, as measured by the Tilburg Frailty Index [15] (TFI score of 5 or more)

These criteria apply for both people living in care homes and people living at home and receiving home care. Older people who move during the follow-up of the study can remain included in the study.

### Intervention

Older people in the intervention group will be offered the Respecting Choices program in addition to their usual care. This program, developed in the US, involves trained nurse facilitators who, in collaboration with physicians, assist residents and their relatives in reflecting on the resident’s goals, values and beliefs and in discussing their health care wishes [11, 16]. This discussion also supports people to identify specific activities and experiences that may contribute to, or detract from, their quality of life. Residents are encouraged to appoint a surrogate decision-maker, and to document their wishes about the care they do or do not want to receive in an advance directive. These wishes can e.g. concern the (non)use of burdensome life-prolonging interventions such as hospitalization or cardio-pulmonary resuscitation. For our study, a nurse practitioner followed the Respecting Choices train-the-facilitators program in the US. She will deliver the training program for the facilitators. These are nursing staff members of Laurens, who will deliver the intervention to the study participants. The intervention concerns two meetings of a facilitator with a participant of 1 h. The content of the communication during these meetings will be structured by the use of interview cards.

### Primary and secondary outcomes

The primary outcome measure is patient activation, which measures patient participation in medical decision making [17]. Secondary outcome measures are quality of life, satisfaction with health care, cost-effectiveness, use of burdensome medical interventions, appointment of a surrogate decision- maker and documentation of care wishes in advance directives.

### Assessment and data collection

At baseline, in personal structured interviews, patient activation will be measured (PAM-13) [17]. The PAM assesses patients’ knowledge, skill and confidence in self- management. Also, generic Health-Related Quality of Life as measured by the SF-12 [18] and satisfaction with health care as measured by the PSQ-18-SF6 [19] will be assessed.

At 12 month follow-up participants will be interviewed again and their competence will be assessed with an adapted version of the Mini-Cog [20]. If participants are competent, the questions of the SF-12 [18], PAM [17], and PSQ-18-SF6 [19] will be asked. We will also ask whether participants completed an advance directive and assigned a proxy decision-maker. In the intervention group, open (qualitative) questions will be asked about how the patient experienced the ACP program.

If the Mini-Cog indicates that participants are no longer competent, a proxy, assigned by the patient at the start of the study, will be approached for a telephone interview. This interview will include the PSQ-18-SF6 [19] and a caregiver activation measure (CAM) [21]. Completion of an advance directive and assignment of a proxy decision-maker by the participant will be assessed. In the intervention group, open (qualitative) questions will be asked about how the relative and the patient experienced the ACP program.

If people pass away during the 12 month follow-up, a proxy, assigned by the patient at the start of the study, will be approached for a telephone interview. This interview will address characteristics of the dying process, quality of dying [16], PSQ-18-SF6 [19], feelings of anxiety and depression of the relative as measured by the HADS [22]. In the intervention group, open (qualitative) questions will be asked about how the relative and the patient experienced the ACP program.

In a medical file study, we will investigate whether people have an advance directive in their medical file (and study its content). Moreover, we will study the medical care people receive during 12 months after inclusion, such as hospitalizations (number, length of stay), use of homecare, palliative care, diagnostic procedures and specific treatments (e.g. medication, mechanical ventilation, resuscitation, chemotherapy, artificial fluids or food, surgery or antibiotics).

Furthermore, an extensive economic evaluation of ACP will be performed from a health care perspective. For the calculation of the health care costs we will distinguish intramural and extramural medical costs. The unit price of the ACP program will be determined with the micro-costing method, which is based on a detailed assessment of all resources used [23]. Therefore, ACP facilitators will register their time investments per individual participant. Costs for inpatient days in care homes will be estimated as real, basic costs per day using detailed administrative information. For the calculation of other medical costs, we will use charges as published in Dutch guidelines as a proxy of real costs [24].

### Sample size

We aim at an overall power of 0.8 (alpha 0.05) to detect a difference of at least 0,5 SD in the Patient Activation Measure. We consider 0,5 SD to be a clinical relevant difference based on the article of Norman et al. [25]. To be able to detect such a difference we need 63 individuals in each arm in a non-clustered study. We used a multiplication factor of (1 + (k-1)*ICC), with k indicating the average cluster size, which is 12, and ICC indicating the intraclass correlation, which is the fraction of total variation in the data that is accounted for by between-group variation. For an ICC of 0.05 this amounts to a multiplication factor of 1.55, and thus 98 (1.55*63) individuals in both the intervention and the control arm.

### Study procedures

**Fig. 1:**
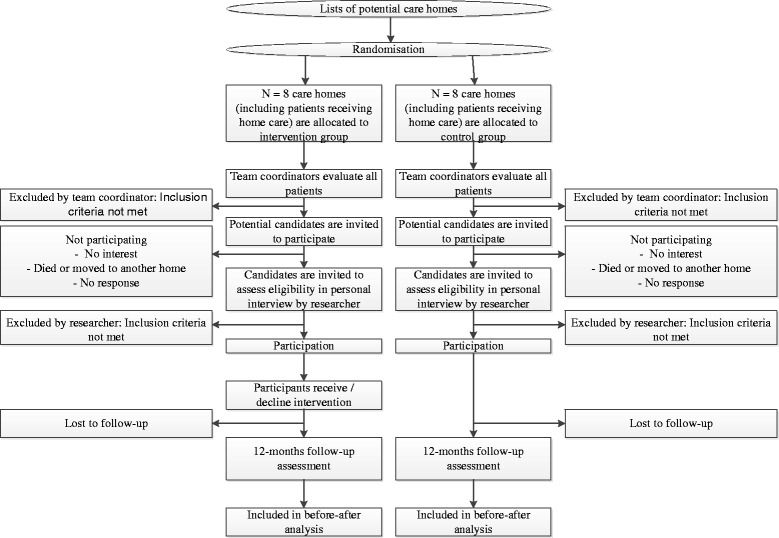
CONSORT flow chart

Staff of the care organisation will use a checklist to assess whether participants - either living in care homes or at home - are potentially eligible for the study (see Fig. 1: CONSORT Flow chart). These people will be informed about the study and will be asked to participate by the research team. Furthermore, in the intervention group they will be invited to visit an informative meeting about the study and the intervention.

People who express an interest in the study, will be interviewed face-to-face by one of the researchers (AO, LJ). During this interview, they will receive additional information about the study. When individuals fulfil the inclusion criteria of the study and want to participate, written informed consent will be obtained. Subsequently, the baseline assessment will be completed and the intervention will be carried out in the intervention group. At 12 months after inclusion in the study, participants will be approached again by the researcher to complete a face-to face follow-up assessment. With written permission of the participants, data will be extracted from medical files with the use of a checklist, 12 months post-inclusion.

The independent Ethics committee of Rotterdam (Medisch Ethische Toetsingscommissie) has given approval for the study to be performed.

### Statistical analysis

Analyses will be performed following the intention-to-treat principle. Descriptive statistics will be used to summarize characteristics of the residents, the care homes and of people receiving home care. Characteristics of participating individuals (age, gender, socio-economic status, educational level) will be compared at baseline between the intervention and control (usual care) group using independent sample t tests and Chi^2^ tests.

Multivariate analysis will be used to compare differences between the intervention group and the control group at baseline and to compare differences in changes after 12 months following the inclusion. Patient characteristics, TFI score, MMSE score and place of residence will be used as covariables. All statistical tests will be considered significant if *p* < 0.05. Missing items will be imputed by the peoples’ own average score if they have completed at least 50 % of the items.

The cost-effectiveness of ACP will be assessed by calculating the incremental cost-effectiveness ratio (ICER), defined as the difference in costs of ACP compared to usual care, divided by the average change in effectiveness, with the patient activation as primary outcome measure [17]. We will perform a sensitivity analysis to assess the stability of the results to changes in costs and effectiveness parameters. Because of the short time horizon, costs and effects will not be discounted.

## Discussion

The presented study measures the costs and effects of ACP in elderly care. Until now, the costs and effects of ACP in Dutch care homes have not been studied in a randomized controlled trial. Currently, ACP is encouraged and several studies showed that ACP is associated with beneficial effects on patient outcomes [6, 7]. However, we do not know whether these beneficial effects apply to frail, older people residing in Dutch nursing homes or living at home and receiving home care. The presented study aims at studying whether offering ACP to older people will improve patient activation, quality of life and satisfaction with care, while reducing futile medical interventions. Trained facilitators will talk with frail, older people about their wishes and preferences concerning medical treatment and care, facilitate timely conversations between participants and their appointed proxy decision-maker and support them in establishing an advance directive. This study will contribute to the implementation of an evidence-based ACP program for older people in The Netherlands. Below, some strengths and limitations of the study will be discussed.

### Strengths and limitations

#### Strengths

First, older people will be offered standardized ACP using the Respecting Choices program [11]. According to Brinkman-Stoppelenburg et al., complex interventions such as e.g. the Respecting Choices program are the most promising ACP programs [6]. Second, this study assesses costs. Until now no formal cost-effectiveness studies of ACP programs in care homes or nursing homes have been carried out. A methodological strength is that we will conduct a cluster randomized control trial which is the most preferred method of studying effects of ACP. Due to this design we will be able to draw conclusions on causal relationships.

#### Limitations

First, selection bias in identifying potential study participants cannot be ruled out. To prevent this as much as possible, we have prepared clear in and exclusion criteria. These criteria are the same in both the intervention arm and the control arm.

Second, follow-up of the respondents is limited to 12 months after inclusion, while the average life expectancy of the study group is 3,7 years [13]. We expect that respondents may need to make important health care decisions within the timeframe of the study, given their frailty, but we may miss important decisions. However, the main goal of the study is to increase patient activation, and such activation is possible without major decisions taking place. Examples of this are conversations with relatives and health care professional about preferences regarding future care.

Third, dropout will be an inevitable limitation of the study. Dropout can occur due to, for example, loss of contact. However, we ask staff members of Laurens to report participants moving house. If people move they can remain included in the study. Dropout can also take place because people are no longer interested in participation. However, we expect that the number of dropouts will be similar in both groups. If participants die during follow-up, one of their relatives will be asked to complete a bereaved carer questionnaire.

Fourth, the extent to which medical files reflect actual care can be questioned. We are not sure that all received treatments will be written down in medical files.

### Opportunities

Realization of the study will contribute to more understanding of the effects of ACP in older, frail people in Europe. We will also gain insight in the costs and cost-effectiveness of ACP, which has rarely been studied until now. Positive outcomes of this study may facilitate the implementation of ACP in the target population of this study, but also in other populations and settings, such as younger people and/or people with advanced diseases. Future research could focus more on opinions and experiences of the patient’s relatives. We hope that our results will encourage debates and discussions about optimal decision making in the last phase of life and will lead to further studies, nationally and internationally.
